# Body composition changes in women with early breast cancer after adjuvant treatment: a systematic review

**DOI:** 10.2340/1651-226X.2025.44707

**Published:** 2025-12-07

**Authors:** Valdemar Mendez, Simone Diedrichsen Marstrand, August Nielsen, Trine Lund-Jacobsen, Caroline Kistorp, Peter Schwarz, Kristian Buch-Larsen

**Affiliations:** Department of Nephrology and Endocrinology, Centre for Cancer and Organ Diseases, Copenhagen University Hospital, Rigshospitalet, Copenhagen, Denmark

**Keywords:** Early breast cancer, chemotherapy, endocrine therapy, body composition, fat mass, lean body mass

## Abstract

**Background and purpose:**

The objective of this systematic review was to establish an overview of changes in body composition as a result of early breast cancer treatment.

**Patient/material and methods:**

Five databases (PubMed, CINAHL, Embase, Web of Science and Cochrane Library) were used for identifying studies and papers. Selection criteria included: > 18 years, early breast cancer stage 0–III and measurement of body composition with either dual X-ray absorptiometry (DXA), magnetic resonance imaging (MRI) or computed tomography (CT). Studies using only bioelectrical impedance were excluded.

**Results:**

A total of 734 studies were screened; 29 studies were full-text reviewed, and 10 studies were included in this systematic review, with a total of *n* = 1,062. Included studies were published from 2018 to 2024. This review found consistent increases in fat mass between 3.3 and 9.2% across the studies. Results for lean body mass were less consistent. Two studies examined visceral fat mass, yet both found statistically significant increases.

**Interpretation:**

This systematic review identified consistent increases in total fat mass and visceral fat across the included studies, regardless of whether the treatment involved chemotherapy, endocrine therapy or a combination of both. In contrast, findings related to lean body mass were considerably less consistent. The results highlight the potential implications following breast cancer treatment and emphasise the importance of metabolic monitoring, diet and exercise to increase quality of life and prevent recurrence. This review also highlights the need for more research on the topic, as the included studies exhibit substantial heterogeneity, making it difficult to draw definitive conclusions.

## Introduction

Globally, breast cancer accounted for 11.6% of all new cancer cases in 2022 and continues to be the most frequent cancer among women worldwide [1]. Metastatic breast cancer accounts for 5–10% of the annual cases, with a median survival rate of 3–4 years [2]. To this, patients with oestrogen receptor (ER)-positive and human epidermal growth factor receptor 2 (HER2)-positive disease have improved survival by year of diagnosis [2]. However, there is a large and growing group of non-metastatic breast cancer survivors, because of early diagnosis and improved treatment [2]. Treatment of non-metastatic breast cancer predominantly consists of surgery and adjuvant therapy, which typically include chemotherapy using a combination of anthracyclines, cyclophosphamide and taxanes [3, 4]. Furthermore, approximately 75% of breast cancers are ER-positive. This is typically treated with antihormonal therapy for 5 years, which aims to reduce the risk of recurrence [4]. However, treatment of breast cancer, including both chemotherapy and antihormonal therapy, is associated with a range of adverse effects. Potential side effects include fatigue [5], vasomotor symptoms [5] reduced bone mineral density (BMD) [6], insulin resistance [7, 8], weight gain [9], neuropathy [10] and more. Furthermore, loss of oestrogen as seen in menopause transition, is associated with accelerated gains in fat mass and loss of lean body mass (LBM) [11], which raises the question of whether antihormonal treatment will have the same consequences. Previous studies demonstrated that reduced LBM, elevated fat percentage and/or fat mass and increased visceral fat mass are negatively correlated with breast cancer progression after adjuvant therapy [12, 13]. Given that these body composition changes are linked to recurrence, mortality and diminished quality of life, understanding their treatment-related causes is of considerable clinical importance. This prompts further investigation into whether the treatments themselves contribute to unfavourable changes in body composition.

*The aim of this systematic review* was to synthesise evidence on how adjuvant chemotherapy and endocrine therapy influence body composition – specifically fat mass, visceral fat and LBM – in women with early breast cancer (EBC).

## Patients/material and methods

### Information sources, strategy and selection process

This review is registered with the PROSPERO International Prospective Register of Systematic Reviews (#CDR42024589810).

**Figure 1:**
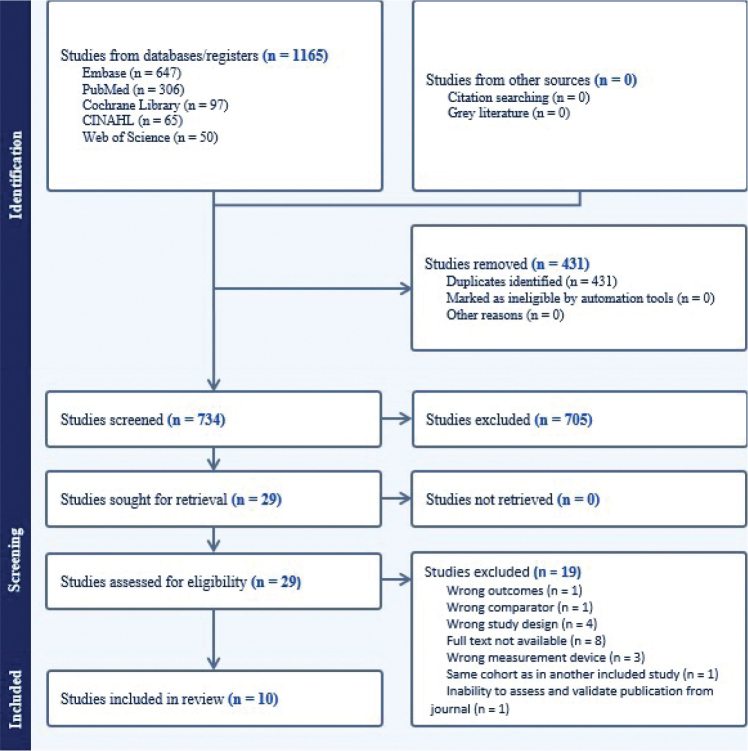
Screening process and flow chart.

The following databases were searched in January 2025 to identify potentially eligible studies: PubMed, CINAHL, Embase, Web of Science and Cochrane Library. Selection criteria included the following: human studies, female participants, > 18 years and early breast cancer stage 0–III. Exclusion criteria included the following: < 18 years, stage 4 cancer (metastatic), animal studies, measurements at only one timepoint and measurement of body composition with technology other than dual X-ray absorptiometry (DXA), computed tomography (CT) or magnetic resonance imaging (MRI), for example, bioelectrical impedance technology. Prospective and retrospective cohort studies and clinical trials observing and measuring body composition before, during and after breast cancer treatment were considered for inclusion. To ensure consistency and scientific quality, only peer-reviewed published studies written in English were included, while grey literature was excluded. To search for literature, a controlled vocabulary (MeSH) and keyword terms were used as well as Boolean operators that matched the database it was searched on. For PubMed the following phrase was used: *(Breast Neoplasms[mh]) OR (‘early breast cancer’[text word] OR ‘early breast carcinoma’ OR ‘early-stage breast cancer’ OR ‘non-metastatic breast cancer’ OR ‘non metastatic breast cancer’)) AND (body composition[mh] OR Body Fat Distribution[mh] OR ‘body composition’ OR ‘fat’ OR ‘lean body mass’ OR ‘visceral fat’).* These search criteria resulted in 734 studies. All articles were imported to Covidence (Covidence systematic review software, Veritas Health Innovation, Melbourne, Australia; available at www.covidence.org) to be screened and reviewed. A primary and secondary reviewer (VM and SM) independently screened and reviewed both the title and abstract of the studies found to decide whether they met the inclusion criteria of this review. Studies that met the selection criteria were included in this review, and excluded articles were recorded in a table of characteristics of excluded studies, with justification. Furthermore, references from full-text articles were examined and reviewed to be included as additional studies. Figure 1 showcases the methodology of narrowing down the included studies.

### Data extraction and analysis

Study outcomes and other data from the included studies were extracted in Covidence. Here, both a tabular and narrative data analysis were conducted by VM and AN and checked for inter-rater reliability. Furthermore, a risk of bias assessment was done with the ROBINS-I V2 tool, and a quality assessment was done with the Mixed-Method Appraisal Tool (MMAT) (version 2018). Quality assessment and risk of bias assessments can be found in Supplementary materials. Analysis was discussed with PS, CK and KBL.

## Results

### Study characteristics

The 10 included studies were published in 10 different peer-reviewed scientific journals between 2018 and 2024. The countries of origin for the studies were Italy [14, 15], Canada [16, 17], The Netherlands [18], Australia [19], France [20], China [21], the USA [22] and Denmark [23]. Only one study was a longitudinal case-control study, where both a study population and a healthy control group underwent measurements at multiple timepoints [18]. Two studies [16, 19] included a healthy control group at baseline but did not assess the healthy control group at follow-up visits, as they did with the patients, to assess changes over time. The remaining studies only included breast cancer patients [14, 15, 17, 21–23]. The majority of the studies were prospective [15, 16, 17, 18–23], following participants before, during and after chemotherapy and/or before and during endocrine treatment. Two of the studies were retrospective [14, 21], one examining the effect of neoadjuvant therapy on the body composition [21] and the other looking at measurements from before, during and after chemotherapy [14]. Study sample sizes ranged from 16 to 361 participants. A total of 1,062 participants were recruited across the included studies. For further detail, see Table 1.

**Table 1:** Summary of methods and aims across included studies.

Authors, year of publication, country										
Longitudinal observational cohort studies										
Recruited patients (withdrawals)	Kirkham et al., 2022. Canada *n* = 34 (0)	van den Berg et al., 2022. The Netherlands *n* = 361 (37)	Burt et al., 2022. Australia *n* = 16 (4)	Mazzuca et al., 2018. Italy *n* = 21 (NA)	Pedersini et al., 2024. Italy *n* = 347 (unknown)	Ginzac et al., 2018. France *n* = 33 (0)	Kirkham et al., 2022. Canada *n* = 94 (0)	Zhang et al., 2024. China *n* = 43 (NA)	Ballinger et al., 2018. USA *n* = 80 (6)	Fredslund et al., 2019. Denmark *n* = 36 (3)
Data collection timepoints	Pre-chemotherapy, mid-chemotherapy, post-chemotherapy, 1 year post-chemotherapy	Pre-chemotherapy, post-chemotherapy, 6 months post-chemotherapy	Pre-chemotherapy, post-chemotherapy	Pre-chemotherapy, during chemotherapy, post-chemotherapy	Pre-aromatase inhibitor treatment, 18 months post	Pre-chemotherapy, 1 month post-chemotherapy, 6 months post-chemotherapy, 3 years post-chemotherapy	Pre trastuzumab therapy, post cycle 4, post trastuzumab therapy	Pre neoadjuvant therapy, post-neoadjuvant therapy	Pre breast cancer treatment, 6 months, 12 months	Pre-chemotherapy, post-chemotherapy, 1 year post-chemotherapy
Aims	To examine changes in body composition among other things, over 12 months of cardiotoxic chemotherapy for early breast cancer	To assess changes in body composition during and after chemotherapy in breast cancer patients compared with age-matched women not diagnosed with cancer	To determine whether insulin sensitivity, cardiovascular risk markers and body composition were perturbed in women treated with chemotherapy for early breast cancer	To examine the association between toxicities and sarcopenia in early breast cancer patient receiving adjuvant chemotherapy	To analyse changes in body composition in early breast cancer patients treated with aromatase inhibitors	To assess the evolution of body composition among postmenopausal breast cancer patients receiving endocrine therapy after standard chemotherapy	To characterise longitudinal cardiac and cardiometabolic effects of trastuzumab, as well as 2 different types of blood pressure lowering medication	To evaluate the effects of neoadjuvant therapy on body composition, bone mineral density and glucose and lipid metabolism	To examine the side effects of different therapy models for breast cancer patients (local therapy, endocrine therapy and chemotherapy with or without endocrine therapy)	To investigate metabolic side effects of adjuvant treatment in breast cancer patients, including body composition
Inclusion criteria	> 18 year. Stages I–III breast cancer. Scheduled to receive trastuzumab-containing and/or anthracycline-containing chemotherapy	> 18 years old. Stages I–III breast cancer. Scheduled to receive adjuvant or neo-adjuvant chemotherapy	> 18 years old. Stages I–III breast cancer. Female. Scheduled to receive adjuvant or neoadjuvant chemotherapy	Stages I–III breast cancer. Adjuvant anthracycline-based adjuvant chemotherapy received for at least 4 cycles	Stages I–III breast cancer. Eligibility to adjuvant treatment with aromatase inhibitors	Stages I–III breast cancer. Postmenopausal women receiving chemo- and endocrine therapy	> 18 years. HER2-positive early breast cancer. Scheduled to receive adjuvant trastuzumab	18–70 years old. Stages II–III breast cancer. Complete neoadjuvant therapy throughout the course	Stages I–III breast cancer or ductal carcinoma in situ. No therapy initiated	> 18 years. Stage I-III breast cancer. Assigned to receive adjuvant chemotherapy.
Methods	Prospective. 4 MRI scans was performed pre-chemotherapy, mid-point of chemotherapy, at the end of chemotherapy, and 1 year after baseline	Prospective. DXA scans were performed before, after and 6 months after chemotherapy for the breast cancer patients. For the control group, 3 scans were done at a similar timeframe. The total cohort included 181 breast cancer patients and 180 women without cancer	Prospective. DXA scans were performed before and after chemotherapy	Retrospective. CT scans performed before, during and after adjuvant chemotherapy were analysed	Prospective. DXA scans were performed before aromatase inhibitor treatment (post-chemotherapy), and after 18 months of treatment	Prospective. DXA scans were performed pre-chemotherapy, 1 month post, 6 months post and 3 years post	Prospective. MRI scans were performed before trastuzumab therapy and repeated after 4 cycles (~3 months) of trastuzumab and at completion of the therapy (~12 months). The cohort was divided into three groups: bisoprolol, perindopril, and placebo	Retrospective. CT scans done before and after neoadjuvant therapy were analysed for changes in body composition	Prospective. DXA scans were performed at baseline before any treatment was initiated, and after 6 and 12 months from enrollment	Prospective. DXA scans were performed prior to, immediately after and 1 year after adjuvant chemotherapy is ended

MRI: magnetic resonance imaging; DXA: dual X-ray absorptiometry; CT: computed tomography.

### Participant characteristics

Participant mean age ranged from 51 to 64 years. None of the studies included only premenopausal women, while one study included only postmenopausal women [20]. Four studies included both pre- and postmenopausal women and reported the ratio [18, 19, 21, 23]. Five studies did not report the menopausal status of the included participants [14, 15–17, 22], but based on the age range/standard deviation (SD) of the studies (Table 2) and the inclusion/exclusion criteria, these studies recruited both pre- and postmenopausal women. However, the distribution of the two was unknown in the studies. All participants across the studies were diagnosed with non-metastatic breast cancer stages I–III at the time of inclusion, as part of the inclusion criteria. Six studies reported the ratio between the stages [14–16, 18, 22, 23]. The remaining four studies did not. However, Ginzac et al. [20] reported the TNM classification of the tumor: pT and pN. The remaining three studies did not report the distribution of cancer stages within the study [17, 19, 21]. For further detail, see Table 2

**Table 2:** Participants’ baseline characteristics across included studies.

Authors, year of publication, country, number of participants													
Main theme	Kirkham et al., 2022. Canada (*n* = 34)	van den Berg et al., 2022. The Netherlands (*n*= 361)		Burt et al., 2022. Australia (*n* = 16)	Mazzuca et al., 2018. Italy (*n* = 21)	Pedersini et al., 2024. Italy (*n* = 347)	Ginzac etal., 2018. France (*n* = 33)	Kirkham et al., 2022. Canada (*n* = 94)*			Zhang et al., 2024. China (*n* = 43)	Ballinger et al., 2018. USA (*n* = 80)	Fredslund et al., 2019. Denmark (*n* = 33)
		EBC (*n* = 181)	CON (*n* = 180)					Placebo (*n* = 30)	Bisoprolol (*n* = 31)	Perindopril (*n* = 33)			
Age, years	Mean (±SD) 51 (± 10)	Median (IQR) 51.8 (46.7; 58.9)	Median (IQR) 53.3 (46.7; 62.3)	Mean (± SD) 53 (± 9)	Median (range) 54 (39–72)	Mean (range) 60 (28–84)	Median (range) 64 (55–75)	Mean (± SD) 51 (± 7)	Mean (± SD) 53 (±10)	Mean (±SD) 50 (±8)	Mean (± SD) 53.5 (± 10,3)	Median (range) 55 (30–74)	Mean (range) 53.8 (36–71)
Menopausal status, n (%)													
Premenopausal	Unknown	103 (58%)	90 (50%)	7 (44%)	Unknown	72 (16.8%)**	0 (0%)	Unknown			17 (40%)	Unknown	20 (61%)
Postmenopausal	Unknown	76 (42%)	89 (50%)	9 (56%)	Unknown	356 (83.2%)**	33 (100%)	Unknown			26 (60%)	Unknown	13 (39%)
Adjuvant treatment, n (%)													
Chemotherapy	34 (100%)	181 (100%)	NA	12 (100%)	21 (100%)	145 (42%)	33 (100%)	94 (100%)			43 (100%) neoadjuvant	32 (40%)	33 (100%)
Endocrine treatment	Unknown	143 (79%)	NA	0 (0%)	0 (0%)	347 (100%)	33 (100%)	0 (0%)			0 (0%)	48 (60%)	29 (88%)
Breast cancer stage, n (%)													
I	4 (12%)	45 (25%)	NA	Unknown	7 (33%)	232 (66.9%)	Unknown	Unknown			0 (0%)	35 (44%)	4 (12%)
II	25 (74%)	110 (61%)	NA	Unknown	11 (52%)	115 (44.1%) (stage ≥II)	Unknown	Unknown			Unknown	26 (33%)	11 (33%)
III	5 (15%)	26 (14%)	NA	Unknown	3 (14%)	Unknown	Unknown	Unknown			Unknown	7 (9%)	16 (48%)
In situ	0 (%)	0 (0%)	NA	Unknown	0 (0%)	Unknown	Unknown	0 (0%)			0 (0%)	12 (15%)	0 (0%)

EBC: early breast cancer; CON: control group.

* VM contacted the corresponding author to obtain the full dataset, as the baseline characteristics are divided into three groups while the results regarding body composition are presented as one group. Furthermore, n for the specific cancer stages is not stated in the article. Authors did not respond.

** Full cohort consisted of 428 EBC patients but only 347 underwent body composition measurements.

### Data gathering and outcome characteristics

Six studies used DXA to examine body composition [15, 18– 20, 22, 23], two used MRI [16, 17] and two used CT [14, 21]. Reported outcomes from the DXA studies included whole body fat mass [15, 18–20], total fat percentage [22, 23], visceral fat [15, 17] and LBM [15, 18–20, 22, 23]. Of the MRI studies, one reported on intermuscular fat, subcutaneous fat and muscle mass in the thigh [16], and the other study reported on visceral fat, subcutaneous fat, intermuscular fat and LBM in the abdominal area [17]. The two CT studies reported on L3 (third lumbar vertebrae) skeletal muscle index [14], cross-sectional area (CSA) [14] and CSA of the pectoralis muscle [21]. All the systemic treatments in the included studies have been in addition to local therapy (surgery and/or radiotherapy).

### Study findings

#### Outcomes on fat mass

Pedersini et al. [15] found, for breast cancer patients treated with 18 months of aromatase inhibitors (AI), a significant increase in fat mass (+7.2%, *p* < 0.01) and visceral fat mass (+18.9%, *p* < 0.01). Van den Berg et al. [18] observed, for breast cancer patients, a significant increase in fat mass from pre-chemotherapy to 6 months post-chemotherapy (+3.3%, *p* < 0.05), whilst no change was observed from pre- to post-chemotherapy, nor in the control group at any timepoint. Nevertheless, the study found that fat mass did not differentially change over time between the two groups (*p* interaction = 0.19). Kirkham et al. [16], found a statistically significant increase in intermuscular fat from pre-chemotherapy to mid-chemotherapy, post-chemotherapy and 1 year following cessation of chemotherapy (+5.5%, +9.2%, +7.6%, *p* < 0.05), but no change in subcutaneous fat of the thigh at any timepoint. Ginzac et al. [20] reported no change in fat mass following 3 years of AI treatment. Ballinger et al. [22] reported an overall increase in fat percentage 12 months after cessation of the respective treatment (+1.8% points, *p* < 0.001). For the specific subgroups the study reported, for cohort A, which had only received local therapy (radiation + surgery), no change in fat percentage; for cohort B, local therapy and AI therapy, an increase in fat percentage (+1.9% points, *p* < 0.001); and, lastly, for cohort C, local therapy and chemotherapy with or without AI treatment, a significant increase in fat percentage (+2.9%-points, *p* < 0.001). Kirkham et al. [17] found in the overall cohort an early significant increase from baseline to trastuzumab cycle 4 in visceral (+7%, *p* = 0.02) and intermuscular fat (+8%, *p* = 0.02), while no change was found in subcutaneous fat. From cycle 4 to completion of trastuzumab therapy, the study observed a significant increase in subcutaneous fat (+6%, *p* = 0.02). Furthermore, from cycle 4 to completion of therapy, visceral and intermuscular fat decreased slightly, though not significantly. However, the reduction was sufficient to negate the statistical difference between baseline and completion of therapy. Burt et al. [19] found no change in fat mass when comparing pre-chemotherapy with post-chemotherapy. Fredslund et al. [23] found, for premenopausal breast cancer patients, a tendency towards increased fat percentage, yet not statistically significant, from pre- to post-chemotherapy (+0.9% points, *p* = 0.06). However, a significant increase was observed when comparing pre-chemotherapy to 12 months post (+2.6% points, *p* = 0.01). For postmenopausal patients the study found no change in fat percentage at any timepoint. For futher data on fat mass, please see Table 3

**Table 3:** Summary of body composition outcomes across included studies.

Authors, year of publication, country													
	Kirkham et al., 2022. Canada (*n* = 34)	van den Berg et al., 2022. The Netherlands (*n* = 361)		Burt et al., 2022. Australia (n = 16)	Mazzuca et al., 2018. Italy (*n* = 21)	Pedersini et al., 2024. Italy (*n* = 347)	Ginzac et al., 2018. France (*n* = 33)	Kirkham et al., 2022. Canada (*n* = 94)	Zhang et al., 2024. China (*n* = 43)	Ballinger et al., 2018. USA (*n* = 80)			Fredslund et al., 2019. Denmark (*n* = 33)
		EBC (*n* = 181)	CON (*n* = 180)							Cohort A (*n* = 15)	Cohort B (*n* = 33)	Cohort C (*n* = 32)	
Type of treatment examined	Chemotherapy	Chemotherapy	NA	Chemotherapy	Chemotherapy	Aromatase inhibitor	Endocrine therapy	Chemotherapy	Neo-adjuvant therapy	Local therapy alone	Anti-estrogen	Chemotherapy with or without anti-estrogen	Chemotherapy
Fat mass	↑ /→	↑	→	→	NA	↑	→	(↑ /→)* /↑	NA	→	↑	↑	↑/→
	(Intermuscular/							(Intermuscular/					(Premenopausal/
	subcutaneous)							subcutaneous)					postmenopausal)
Lean body	→	→	→	→	→	↓	↑	→	↓	→	↑	↓	→
mass													
Visceral	NA	NA		NA	NA	↑	NA	↑/→*	NA	NA			NA
fat mass													

↑/↓ indicates significant increase/decrease with a p value of ≤ 0.05.

→ indicates no significant change.

* Significant change from baseline to 4th trastuzumab cycle, but no change at the completion of therapy.

### Outcomes on lean body mass

Pedersini et al. [15] found a significant decrease in LBM after 18 months of AI treatment (−3.1%, *p* < 0.01). Van den Berg et al. (18] observed, for breast cancer patients, a significant increase in LBM from pre- to post-chemotherapy (+2.1%, *p* < 0.05) and a significant decrease in LBM from post-chemotherapy to 6 months post-chemotherapy (−2.1%, *p* < 0.05); hence, it normalised. In the control group, no change in LBM was observed. The study also reported that the change in LBM over time in the patient group differed from LBM change over time in the control group (*p* interaction < 0.01). Kirkham et al. [16] observed no change in LBM at any timepoint. Ginzac et al. [20] found a significant increase in LBM from pre-chemotherapy to post 3 years of AI treatment (+1.5 ± 3.2 kg, *p* = 0.0083). Ballinger et al. [22] observed an overall significant reduction in LBM 12 months after cessation of the respective treatment (−2.6%, *p* = 0.04). For the specific subgroups the study reported, for both cohorts A and B, no change in LBM. In cohort C, the study found a significant reduction in LBM (-3.6%, *p* = 0.007). Kirkham et al. [17] found in the overall cohort, from baseline to trastuzumab cycle 4, no change in muscle volume. From cycle 4 to completion of trastuzumab therapy, the study observed a significant decrease in muscle volume (−2%, *p* = 0.008). Burt et al. [19] observed no change in LBM from pre- to post-chemotherapy. Zhang et al. [21] found a significant reduction in CSA at the pectoralis muscle following neoadjuvant therapy (−9.3%, *p* < 0.001). Mazzuca et al. [14] found no changes in CSA or muscle index at L3 from baseline to during and after chemotherapy. Fredslund et al. [23] found, for both pre- and postmenopausal breast cancer patients, no changes in LBM from pre-chemotherapy to post-chemotherapy as well as 12 months post-chemotherapy. Please see Table 3 for further detail on LBM.

## Discussion and conclusion

A concise summary of the findings indicates that adjuvant cancer therapy is associated with an increase in total fat mass while exerting no significant effect on LBM in female early breast cancer patients. Seven of the 10 included studies assessed the effect of chemotherapy on body composition [14, 16–19, 21, 23]. Data from these studies indicate that chemotherapy alone adversely affects fat distribution, as evidenced by an overall increase in fat mass following the treatment, although not all studies found significant changes. The increases reported were between 3.3 and 9.2%. Regarding muscle mass, only two studies [17, 21] reported a decrease in muscle volume, while one study found an increase [20] and the remaining studies did not find any significant changes. It is noteworthy that the two studies distinguishing between pre- and postmenopausal women [18, 23] tended to report more adverse changes in breast cancer treatment outcomes among premenopausal women. This is in line with findings from other studies that investigated the role of menopausal status on experienced symptoms after adjuvant therapy, for example, sleep and hot flashes [24, 25].

Three studies examined the effect of endocrine therapy as the sole systemic treatment [15, 20, 22]. Here the results were not so streamlined. Pedersini et al. [15] found in his cohort of 347 participants a significant decrease in LBM and significant increases in total fat mass and visceral fat mass after 18 months of AI treatment. Meanwhile, Ginzac et al. [20] surprisingly found a significant increase in LBM after 3 years of endocrine therapy as well as no change in fat mass. The increase in LBM was mainly constituted by those who were obese prior to inclusion, and the authors suggest that body water may have played a role, as they measured body water with bioimpedance and found a 0.8 kg ± 2.5 increase (*p* = 0.08) from baseline to 3 years post-endocrine therapy. Lastly, Ballinger et al. (22] found in cohort B (local treatment + AI therapy), no change in LBM but an increase in fat percentage. Overall, it is difficult to draw any firm conclusions regarding the effects of endocrine therapy on body composition, but the findings from the study of Pedersini et al. [15] ought to be given the most weight, primarily because of the large cohort size.

Ballinger et al. [22] also assessed chemotherapy with or without endocrine therapy (15/32 participants got both therapies) 12 months post diagnosis. The study found convincing results of an increase in body fat percentage and decrease in LBM, suggesting a combination of both chemotherapy and endocrine therapy has an additive negative effect on the body composition compared to the treatments taken in isolation.

The changes found in the included studies may reflect treatment-induced metabolic dysregulation and decreased physical activity. Ultimately, these changes increase the risk of recurrence and, equally important, diminish the health-related quality of life, which has been reported to be significantly lower among breast cancer patients compared to the general population in Denmark [26]. This emphasises the need for exercise and nutrition interventions post-treatment, preferably multimodal exercise and diet programmes, which have been proven to be the most effective interventions to reduce fat mass, fat percentage and body weight and preserve LBM in women with breast cancer [27].

A notable limitation of the included studies is the general absence of control groups. Six of the included studies had no control group included at any time point [14, 15, 17, 20–22]. Three studies included a control group at baseline [16, 19, 23], but only breast cancer patients were invited for follow-up assessments (6/12 months) post-treatment. This methodological limitation substantially restricts the conclusions that can be drawn concerning changes over time, although it could be argued that, because no differences were found between breast cancer patients and controls prior to chemotherapy, any changes observed over the short follow-up time are likely attributable to the direct or indirect effects of cancer therapy, rather than to the cancer itself or pre-diagnostic risk factors. Only one of the included studies in this systematic review had a parallel control group that was followed in the same time frame as the breast cancer patients [18].

Another limitation is a lack of body water assessments in addition to the LBM results in the studies using DXA scans, as body water will be showcased via LBM. None of the studies reported dry lean muscle. As already mentioned, Ginzac et al. [20] found a significant increase in LBM as well as an increase in body water, although not significant. Nevertheless, the authors suggest that the increase in body water may have been a confounder for the increase they found in LBM. Furthermore, van den Berg et al. [18] found an initial increase in LBM from pre- to post-chemotherapy which then returned to baseline values 6 months post-treatment. The study suggests that the initial increase may plausibly have been because of an increase in body fluid. They did not assess body water in the study but refer to a study showcasing an association between fluid retention and docetaxel [28], which is included in a variety of chemotherapy regimens. This supports their hypothesis that the increase found in LBM post-chemotherapy is attributed to an increase in body water because of fluid retention induced by the treatment. Although DXA scans are considered the golden standard for body composition measurements, the validity of the LBM results would have been further improved by adjusting for body water or measuring and reporting it. This methodological limitation is primarily limited to the studies using DXA, as CT and MRI are less affected by hydration status [29].

Lastly, it is noteworthy that of the 10 included studies, two studies did not have changes in body composition as their primary outcome [17, 23]. In these studies, body composition was assessed as a secondary outcome; hence, the protocols were not specifically designed to evaluate the effect of adjuvant breast cancer therapy on body composition. All in all, this review showcases the need for more research on this topic. The protocols of the included studies in this review are very different in many regards, which makes it difficult to make valid conclusions. This includes a big variety in study design and participant characteristics, for example, age: 28–84 years old, the number of participants: *n* = 16–361, menopausal status; assessment timepoints; type of breast cancer treatment; control groups and body composition measurement device used. Future trials should adopt harmonised protocols, integrate imaging-based and metabolic assessments and consider stratification by menopausal status to clarify treatment-related body composition trajectories.

## Conclusion

The most consistent findings in this systematic review relate to fat mass and visceral fat, where the included studies predominantly reported an increase (3.3–9.2%) following the respective breast cancer treatment, with only a few exceptions. The results regarding LBM were less consistent, with most studies reporting no significant changes, while others observed both decreases and increases in LBM after breast cancer treatment. To accommodate these potential implications, increased focus on diet and exercise among the patients is needed as well as increased metabolic monitoring by physicians. Furthermore, this review has highlighted the need for more studies to draw more validated conclusions on the effect of different breast cancer treatments, if any, on the body composition.

## Supplementary Material



## Data Availability

No primary data were collected for this study. All data underlying this systematic review are extracted from previously published studies. Extracted data and materials used in screening and analysis are available from the corresponding author upon request.
